# The complete mitochondrial genome of *Ophiocordyceps gracilis* and its comparison with related species

**DOI:** 10.1186/s43008-021-00081-z

**Published:** 2021-10-20

**Authors:** Aifeire Abuduaini, Yuan-Bing Wang, Hui-Ying Zhou, Rui-Ping Kang, Ming-Liang Ding, Yu Jiang, Fei-Ya Suo, Luo-Dong Huang

**Affiliations:** 1 College of Life Science and Technology, Xinjiang University, Urumchi, 830046 China; 2 CAS Key Laboratory for Plant Diversity and Biogeography of East Asia, Kunming Institute of Botany, Chinese Academy of Sciences, Kunming, 650201 China; 3 Food Crops Research Institute, Yunnan Academy of Agricultural Sciences, Kunming, 650205 China; 4 College of Life Science and Technology, Guangxi University, Nanning, 530004 China; 5 Guangxi Research Center for Microbial and Enzyme Engineering Technology, Guangxi University, Nanning, 530004 China

**Keywords:** Mitochondrial genome, Phylogenetic analysis, *Ophiocordyceps gracilis*, Ophiocordycipitaceae

## Abstract

In this study, the complete mitochondrial genome of *O. gracilis* was sequenced and assembled before being compared with related species*.* As the second largest mitogenome reported in the family Ophiocordycipitaceae, the mitogenome of *O. gracilis* (voucher OG201301) is a circular DNA molecule of 134,288 bp that contains numerous introns and longer intergenomic regions. UCA was detected as anticodon in tRNA-Sec of *O. gracilis*, while comparative mitogenome analysis of nine Ophiocordycipitaceae fungi indicated that the order and contents of PCGs and rRNA genes were considerably conserved and could descend from a common ancestor in Ophiocordycipitaceae. In addition, the expansion of mitochondrial organization, introns, gene length, and order of *O. gracilis* were determined to be similar to those of *O. sinensis*, which indicated common mechanisms underlying adaptive evolution in *O. gracilis* and *O. sinensis*. Based on the mitochondrial gene dataset (15 PCGs and 2 RNA genes), a close genetic relationship between *O. gracilis* and *O. sinensis* was revealed through phylogenetic analysis. This study is the first to investigate the molecular evolution, phylogenetic pattern, and genetic structure characteristics of mitogenome in *O. gracilis*. Based on the obtained results, the mitogenome of *O. gracilis* can increase understanding of the genetic diversity and evolution of cordycipitoid fungi.

**Supplementary Information:**

The online version contains supplementary material available at 10.1186/s43008-021-00081-z.

## INTRODUCTION

Mitochondrion is a semi-autonomous organelle with its own genetic material and genetic system, and it has the ability of independent replication and inheritance. The mitochondrial genome is characterized by a high copy number, rapid evolution, and conservative genetic function (Burger et al. 2003; Bullerwell and Lang 2005; Nadimi et al. 2016). Within ascomycetes, mitochondrial DNA (mtDNA) is often circular and almost all mitochondrial genes are encoded by the same DNA strand (Sandor et al. 2018). Fungal mitogenome normally consists of 14 protein-coding genes (PCGs) including three ATP-synthase subunits, seven NADH dehydrogenase subunits, one complex III (cytochrome c oxidoreductase), and three complex IV (cytochrome c oxidase subunits). Because there are small and large ribosomal RNAs and a set of tRNA genes in the fungal mitogenome (Johnston and Williams 2016; Li et al. 2019b; Wang et al. 2020a), mtDNA has been widely utilized in analyses of molecular evolution, phylogenetic pattern, conservation genetics, population genetics, and genetic structure analyses of fungi (Zhang et al. 2015a; Nadimi et al. 2016; Sandor et al. 2018).

Owing to the efforts of the Fungal Mitochondrial Genome Project (FMGP), many complete mitogenomes of fungi have been sequenced (Paquin et al. 1997; Husami et al. 2020). Regarding the mitochondrial genomes of Ophiocordycipitaceae (Hypocreales), the nine species thereof have been reported and attracted considerable attention (Wang et al. 2016, 2018; Zhang et al. 2015b, 2017a,b; Huang et al. 2017; Kang et al. 2017; Li et al. 2020a; Zhang and Zhang 2020). Research on the Ophiocordycipitaceae mitogenome provided new insights into the systematics and evolution of entomopathogenic fungi, with the size, structure, order, and content of mitogenomes from these species being highly variable. The mitogenome content is typically conserved, but the mitogenome size and gene synteny still vary (Li et al. 2020b). Thus far, a detailed comparison of the mitogenome information has not yet been elucidated, which limits the overall understanding of the phylogenetics and adaptive evolution of Ophiocordycipitaceae species.

Among Ophiocordycipitaceae, *Ophiocordyceps* is morphologically diverse and is the largest genus of the entomopathogenic fungi (Sung et al. 2007). *Ophiocordyceps sinensis* is well-established for its biological functions against diseases and health benefits in humans, and it is extensively used in traditional Chinese medicine (Marioni et al. 2008; Liang 2018). Of note, the bioactive compounds of *O. gracilis* and *O. sinensis* are almost the same such as D-Mannitol, polysaccharide, adenosine, amino acids, and mannitol. This presents common features for efficient utilization and research into the aforementioned *Ophiocordyceps* species. Environment heterogeneity studies have revealed that *O. gracilis* grows in coniferous forests at 1116–2076-m altitude and is widely distributed in Russia, Guatemala, Mexico, Norway, Denmark, France, Nepal, India, Mongolia, Kazakhstan, and China (Sung et al. 2007). *Ophiocordyceps sinensis* parasitizes various ghost moth larvae (Lepidoptera: Hepialidae) and occurs in more diverse habitats (alpine meadow and shrub) in the Qinghai-Tibetan plateau at 3000-m or higher altitude (Li et al. 2011). Hence, habitats can be employed as a useful feature to distinguish *O. gracilis* and *O. sinensis*.

Initially, *O. gracilis* has been referred to as *Xylaria gracilis*, which belongs to Xylariaceae. In 1883, the name was revised to *Cordyceps gracilis*. In the recent phylogenetic classification of insect pathogens of clavicipitaceous fungi, *O. gracilis* (voucher number EFCC 3101, EFCC 8572) was assigned to the *O. ravenelii* clade and was clustered with *O. heteropoda.* However, *O. gracilis* (voucher number OSC 151906) is similar to *Ophiocordyceps* sp. (voucher number OSC151907) within the *O. ravenelii* clade (Sung et al. 2007; Quandt et al. 2014), which suggests that *O. gracilis* is not monophyletic. The *O. gracilis* representative vouchers were clustered in different clades and could have resulted in a case of mistaken identity. The morphological characteristics and small number of genes of *O. gracilis* can be affected by the environment, and difficulties exists in accurately identifying this species using traditional taxonomic methods. Accordingly, further studies are needed to verify the phylogenetic position of *O. gracilis*.

The fungus *O. gracilis* produces compounds with numerous biological and pharmacological properties; yet there is a scarcity of genome and genetic information thereof to clarify the evolutionary relationship. In this study, the complete mitogenome of *O. gracilis* (voucher OG201301) was assembled and annotated. The content, structure, and gene order of the mitogenome of *O. gracilis* were analyzed to deepen the understanding of its mitogenome structure. Based on the characteristics of nine fungal mitogenomes of Ophiocordycipitaceae, the differences between them were compared. Meanwhile, a detailed comparative mitogenomic analysis was conducted on *O. sinensis*, which is a typical entomopathogenic fungus and the largest mitogenome in Ophiocordycipitaceae. Ultimately, phylogenetic analysis adopting complete mitochondrial data provides a theoretical basis for the conservation and sustainable utilization of *O. gracilis* resources and furthers the current understanding of Hypocreales fungi in terms of systematics and evolutionary biology.

## MATERIALS AND METHODS

### Sampling, DNA extraction, and NGS sequencing

*O. gracilis* voucher OG201301 was collected from the Altai Mountains of Xinjiang, China. The asexual strain of OG201301 was isolated as described in a previous study (Wang et al. 2020b). The voucher OG201301 was stored in the fungarium of our laboratory at Guangxi University, and the culture (KUMCC 3001) isolated from that voucher was deposited in the public Culture Collection of the Kunming Institute of Botany (KUMCC). Axenic living cultures were used for genomic DNA extraction using a Plant Genomic DNA Extraction Kit (Takara, Biotech Co., Beijing, China). A total of 5 µg micrograms of genomic DNA from *O. gracilis* was sheared into 270 bp fragments as the DNA library. The library was carried on the Illumina HiSeq 4000 platform for paired-end 150 bp sequencing by BGI Biotechnology Co. (Shenzhen, China). The generated DNA raw data was stored in the NCBI Short Read Archive (SRA) under accession number PRJNA627096.

### De novo assembly and annotation of the mitogenome

To eliminate adaptor sequences, reads with low-quality sequences, and unknown nucleotides, the raw reads were filtered after sequencing. SPAdes 3.9.0 was used to assemble high-quality clean reads (Bankevich et al. 2012). The assembled contig sequences were aligned using BLAST against the Nr database to screen out mitochondrial contigs. One contig of mitochondrial DNA was identified on the basis of sequence similarity to other Ophiocordycipitaceae mitogenomes and the contig gaps were filled through sequence extension and iterative mapping method by PRICE (Paired-Read Iterative Contig Extension) (Ruby et al. 2013). Finally, mitogenome annotation was performed using methods proposed by Li et al. (2019b). The mitogenome was automatically annotated using the MFannot tool (https://megasun.bch.umontreal.ca/cgi-bin/RNAweasel/RNAweaselInterface.pl) based on the mold mitogenome genetic code (Valach et al. 2014). Subsequently, the UGENE ORFs finder was adopted to identify protein-encoding genes (Okonechnikov et al. 2012). The analysis of tRNA and rRNA genes was conducted using tRNAscan-SE 2.0 (Lowe and Chan 2016) and RNAmmer 1.2 Server (Lagesen et al. 2007), respectively. For the gene-containing introns, exonerate software was applied to compare the amino acid sequences of the near-source species to determine the boundaries and lengths of the introns. The introns were detected by RNAweasel (http://megasun.bch.umontreal.ca/cgi-bin/RNAweasel/RNAweaselInterface.pl).

### Sequence analysis

The complete mitogenome sequence along with gene annotations of *O. gracilis* was deposited in the GenBank database under the accession numbers of MT371080, while the physical genome map was drawn using the online software GenomeVx (Conant and Wolfe 2008). The Unipro UGENE was employed to analyze all mitogenomes for the based composition thereof. The following formulas were employed to access mitogenome strand asymmetry: A–T skew = [A–T]/[A + T] and G–C skew = [G–C]/[G + C]. The codon usage was analyzed and visualized using MEGA 7.0 (Kumar et al. 2016). The Mauve 2.3.1 was applied to determine the collinearity of mitogenomes between *O. gracilis* and eight other fungi from Ophiocordicipitaceae (Darling et al. 2004). Subsequently, Circoletto was applied to identify the interspersed repeats through the *O. gracilis* and *O. sinensis* mitogenomes, the E-value of which was 10^−5^ (Darzentas 2010). The repeated sequences were detected by REPuter, in which the default parameters were used (Kurtz et al. 2001). The tandem repeats were located using the Tandem Repeats Finder (https://tandem.bu.edu/trf/trf.basic.submit.html), and simple sequence repeats (SSRs) were distinguished using the online MIcroSAtellites classification tool (Beier et al. 2017).

### Phylogenetic analysis

To investigate the phylogenetic position of *O. gracilis* in the family Ophiocordicipitaceae, a phylogenetic tree of 24 Hypocreales fungi premised on the allocated sequential gene set (14 core PCGs + rps3 + 2 RNA genes) was constructed. For the analysis of homologous gene set sequence, TBtools was used to perform gene sequence extraction and local blast alignment (Chen et al. 2020). The mitochondrial gene set was aligned using RAxML v8.0.0 and MAFFT v7.037 to construct a phylogenetic tree (Stamatakis 2014; Katoh et al. 2017) for which the Bayesian inference (BI) method was adopted. MrBayes v.3.1.2 was applied to conduct BI analysis for three million generations using the most appropriate model, with 1000 bootstrap replicates (Ronquist et al. 2012).

## RESULTS AND DISCUSSION

### Mitogenomic characterization of *Ophiocordyceps gracilis*

**Fig. 1:**
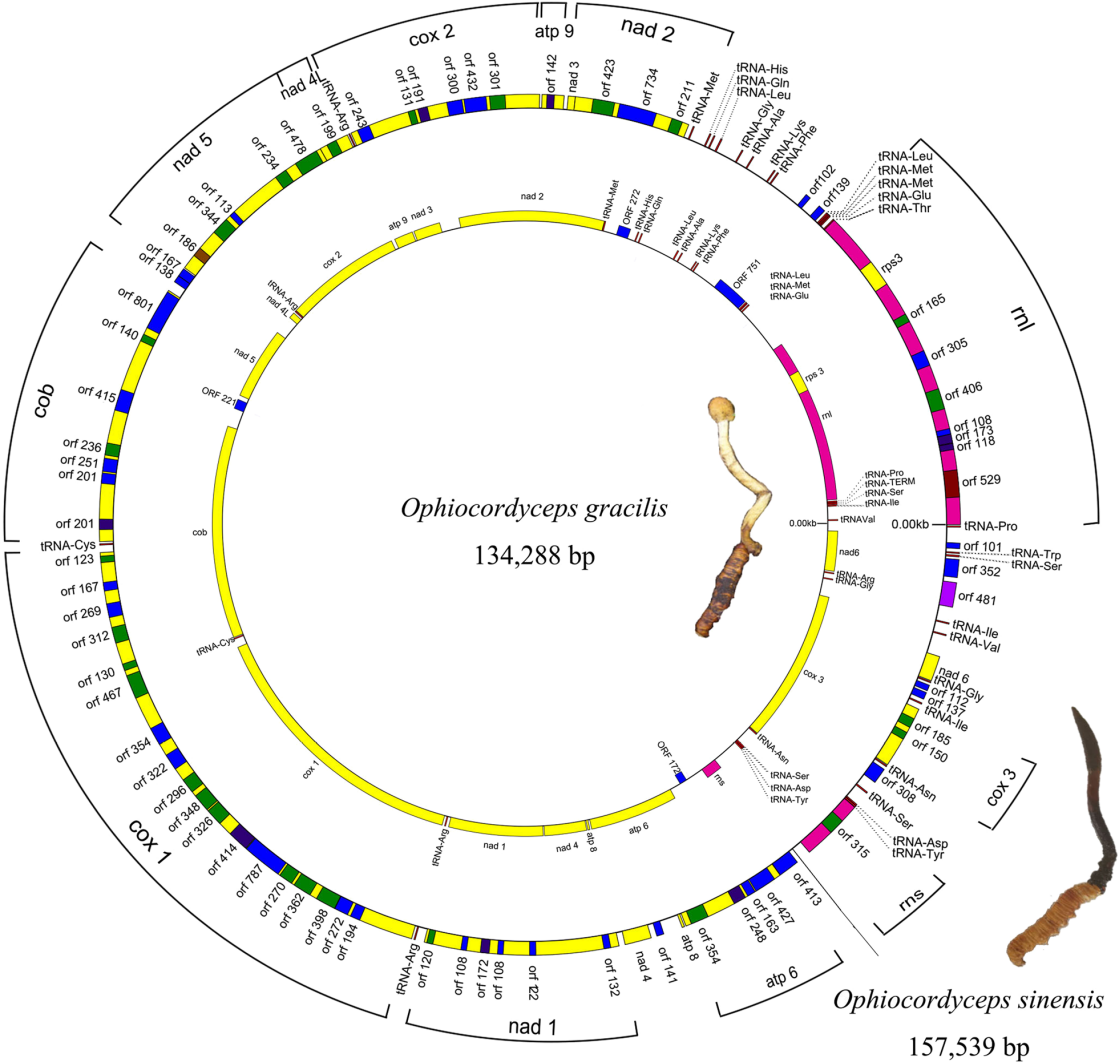
Mitogenome map of *Ophiocordyceps gracilis* and *O. sinensis*. The outer layer represents *O. sinensis* obtained from Kang et al. (2017); the inner layer represents *O. gracilis* from this study. The genes are shown in different color blocks

The complete mitogenome of *O. gracilis* was assembled as a circular molecule 134288 bp long (Fig. 1; Additional file 2: Table S1), and it was the second largest mitogenome reported among the Ophiocoedycipitaceae (Table 1). In general, mitogenomic coding is associated with the mitochondrial translation apparatus, electron transport, and oxidative phosphorylation (Burger et al. 2003).

**Table 1:** Comparison on mitogenomes among nine Ophiocordycipitaceae species

Species		*Ophiocordyceps sinensis*	*Ophiocordyceps gracilis*	*Hirsutella thompsonii*	*Hirsutella rhossiliensis*	*Hirsutella vermicola*	*Hirsutella minnesotensis*	*Tolypocladium ophioglossoides*	*Tolypocladium inflatum*	*Purpureocillium lilacinum*
GenBank accession no		KY622006	MT371080	MH367296	KU203675	KY465721	KR139916	KX455872	KY924883	MN635609
All length (bp)		157539	134288	65332	62483	53793	52245	35159	24973	23495
PCGs	Number	15	15	15	15	15	15	15	15	15
	Length (bp)	14913	15996	15759	14571	14925	15171	14406	14427	14358
	Ratio (%)	9.47	11.91	24.12	23.32	27.75	29.04	40.97	57.77	61.11
tRNA	Number	27	24	27	26	25	25	25	25	22
	Length (bp)	2010	1813	2007	1922	1860	1863	1869	1866	1657
	Ratio (%)	1.28	1.35	3.07	3.08	3.46	3.57	5.32	7.47	7.05
rRNA	Number	2	2	2	2	2	2	2	2	2
	Length (bp)	6558	5253	5145	7067	5163	4898	4694	4569	4562
	Ratio (%)	4.16	3.91	7.88	11.31	9.60	9.38	13.35	18.30	19.42
Introns	Number	54	49	13	9	7	13	6	1	1
	Length (bp)	106554	82380	23972	12477	15549	17123	7319	1690	1638
	Ratio (%)	67.64	61.35	36.69	19.97	28.91	32.77	20.82	6.77	6.97
ORF	Number	73	4	18	8	13	16	4	1	0
Intergenic regions	Length (bp)	29043	30299	20010	28081	17899	14637	8230	3730	2523
	Ratio (%)	18.44	22.56	30.63	44.94	33.27	28.02	23.41	14.94	10.74

The PCGs of *O. gracilis* included 15 PCGs related to oxidative phosphorylation [NAD dehydrogenase (nad1-6 and nad4L), cytochrome oxidases (cox1-3), cytochrome b (cob), and three ATP synthases (atp6, atp8 and atp9), ribosomal protein S3 (rps3)], 4 hypothetical proteins (orf751, orf272, orf172 and orf221), 2 ribosomal RNA genes (rns and rnl), and 24 transfer RNA genes (Additional file 2: Table S1). In the mitogenome of *O. gracilis*, the length of 15 PCGs varied from 147 bp (atp8) to 19,137 bp (cox1). The total length was 15,996 bp, which accounted for 11.91% of the mitogenome. Being 1.35% of the mitogenome, the length of tRNA was 1813 bp, while the length of rRNAs was 5253 bp, which was 3.91% of the mitogenome. Further, the length of intergenic regions (IRs) was 30,299 bp, which accounted for 22.56% (Table 1). Thus, the generated length of *O. gracilis* varied through 15 PCGs, which contributed to mitogenome expansion. The assumption was that the protein-coding regions could be additional factors for the large size of the fungal mitochondria.

The GC concentration of the mitogenome differs among species, which may be affected by mutation bias, selection, and biases of reconstitution-related DNA repair (Li et al. 2018). The nucleotide composition of *O. gracilis* was biased towards AT (71.35%) and the GC content was 28.65%. According to the second parity rule of Chargaff (A–T and G–C within one strand), *O. gracilis* was maintained at the same frequency (Chen et al. 2014), where an observation could be made that GC and AT skews therein were positive with AT-skew < 1 (Additional file 2: Table S1).

Aside from nad4 that was initiated with ATC (Table 2), PCGs were initiated with typical ATG start codons. The remaining PCGs of atp6, cox3, and nad6 start codons were TTA. The majority of PCGs were terminated by typical TAA codons, except for rps3, nad2 and cox1, with two genes terminal codons being TAG. All protein and RNA genes were encoded in positive strands except for orf172 (Additional file 2: Table S1). Similarly, the rps3 coding ribosomal protein S3 was included in rnl (Fig. 1 and Additional file 2: Table S1). Only tRNA-Met was connected to tRNA-Leu, and the longest distance was 3462 bp (tRNA-Glu and rnl), with 8 tRNAs being on both sides of rnl (Additional file 2: Table S1). This result indicates that the mitogenome of *O. gracilis* has non-continuous segments, abundant introns, and lack of overlapping genes (Additional file 2: Table S1).

**Table 2:** PCGs and rRNA genes composition of *Ophiocordyceps gracilis* *mitogenome*

Genes	Location	Start/stop codon	Introns number	Introns length	Exon length	Strand	Coding sequence percentage (%)
rnl	1655–13038	–	5	7261	4123	+	36.22
rps3	9424–10875	ATG/TAG	0	0	1452	+	100
nad2	28088–38134	ATG/TAG	5	8112	1935	+	19.26
nad3	39492–41342	ATG/TAA	1	1437	414	+	22.37
atp9	41459–42766	ATG/TAA	1	1083	225	+	17.20
cox2	43009–51117	ATG/TAA	4	7356	753	+	9.29
nad4L	51358–51780	ATG/TAA	0	0	423	+	100
nad5	53097–58109	ATG/TAA	3	2958	2055	+	40.99
cob	60382–75098	ATG/TAA	8	13544	1173	+	7.97
cox1	75840–94976	ATG/TAG	10	16566	2571	+	13.43
nad1	95433–101968	ATG/TAA	3	5426	1110	+	16.98
nad4	102050–104962	ATC/TAA	1	1449	1464	+	50.26
atp8	105038–105184	ATG/TAA	0	0	147	+	100
atp6	105309–111399	TTA/TAA	2	5317	774	+	12.71
rns	114044–115173	–	0	0	1130	+	100
cox3	118592–129212	TTA/TAA	5	9820	801	+	7.54
nad6	131019–133768	TTA/TAA	1	2051	699	+	25.42

Selenocysteine (Sec) was the 21st discovered amino acid, and UGA is the dominant Sec codon in use (Vargas-Rodriguez et al. 2018); the Sec-UGA codon is a controversial stop codon (Serrão et al. 2018); In *O. gracilis*, UCA is an anticodon in tRNA-Sec, which indicates that UCA-anticodon is involved in the specificity and adaptive evolution thereof.

**Fig. 2:**
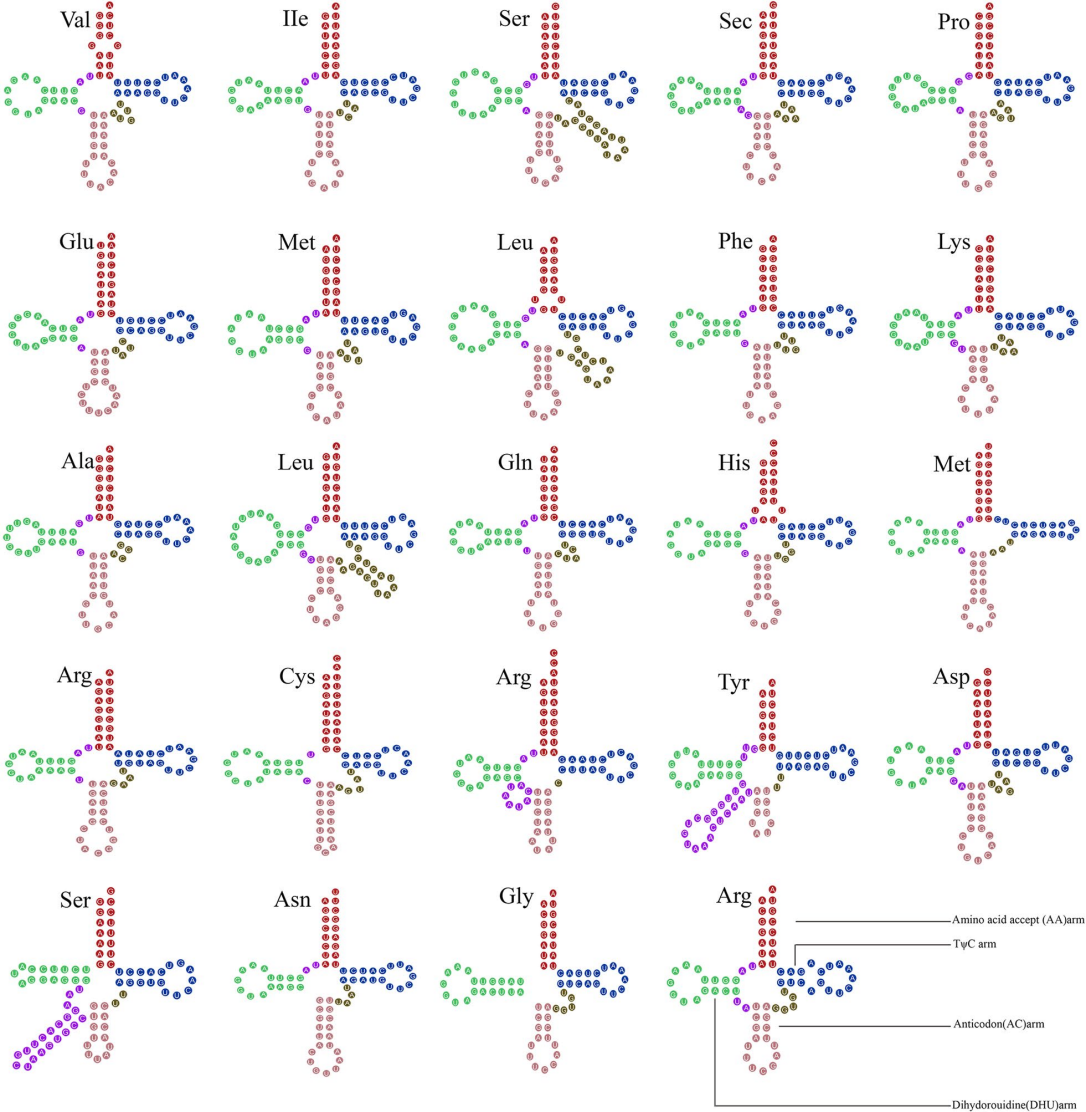
Putative secondary structures of tRNAs from *Ophiocordyceps gracilis* mitogenomes. The amino acid accepter (AA) arm is shown in red; the TψC arm is shown in blue; the anticodon (AC) arm is shown in pink, and the dilhydoroudine (DHU) arm is shown in green

The tRNA had the same significance during translation as mRNA and proteins (Kirchner and Ignatova 2014). In *O. gracilis*, 24 tRNAs genes in the mitogenome encoded the 20 standard amino acids, while tRNA-Trp and tRNA-Thr were not predicted, with a range of 71–87 bp (Additional file 3: Table S2). The difference in the predicted tRNA secondary structure can be ascribed to the different analysis methods adopted. In addition, 19 tRNA exhibited typical clover-leaf secondary structure, i.e., tRNA-Ser, tRNA-Leu, and tRNA-Tyr with a variable loop. All tRNAs exhibited typical clover-leaf secondary structure, and the remaining mismatched bases had non-canonical G–U pairs (Fig. 2). In accordance with the analysis of the secondary structure in fungi, the variations in the numbers of extra arms may cause differences in tRNA length. Further, in *O. gracilis*, there was a lack of tRNA-Thr and tRNA-Trp, while there was one less tRNA-Met and one more tRNA-Arg compared to other fungi (Additional file 4: Table S3).

### Comparative mitogenomics of Ophiocordycipitaceae fungi

**Fig. 3:**
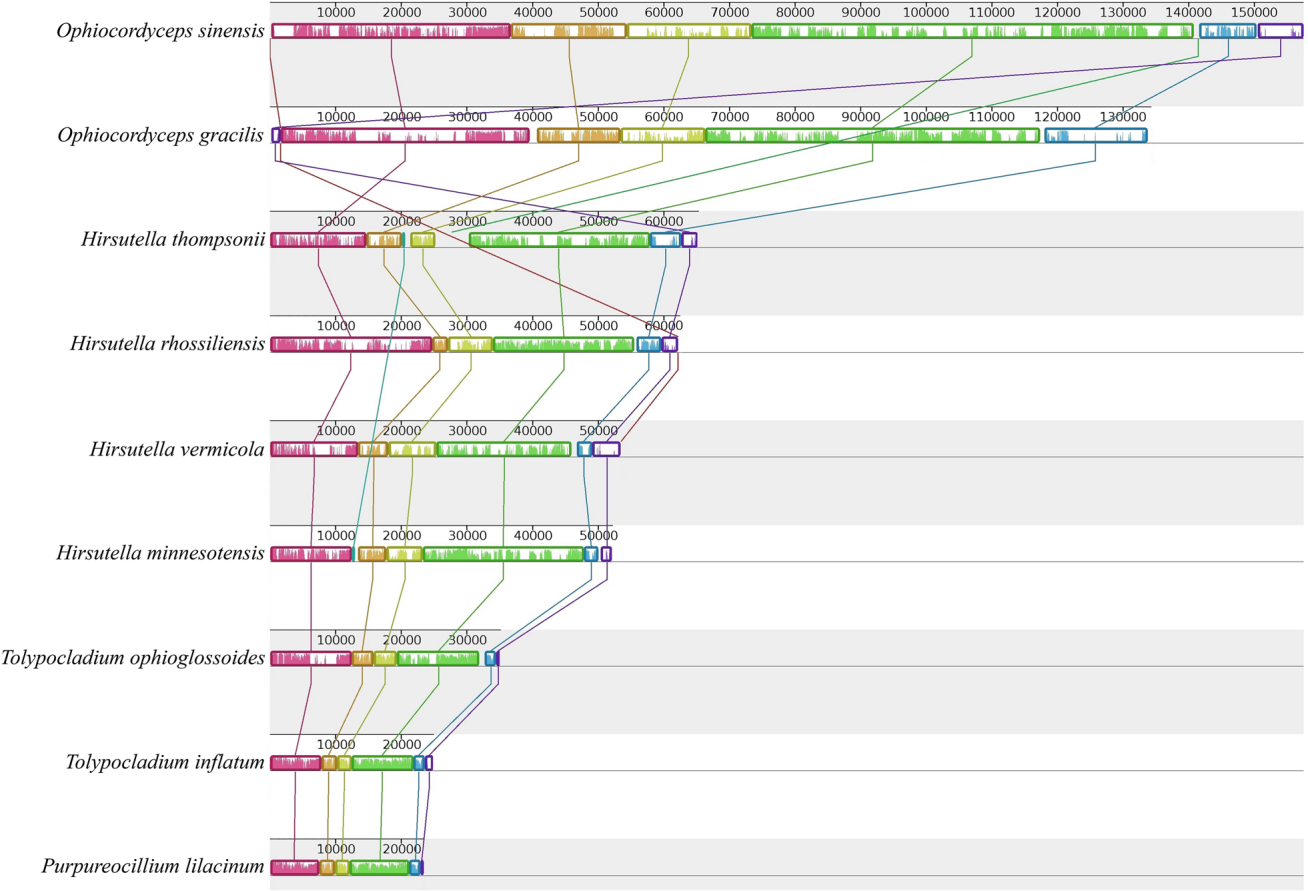
Collinearity analysis of nine mitogenomes from Ophiocordycipitaceae. Homologous regions between different mitogenomes are represented by the same color blocks linked by lines

The nine published fungal mitogenomes of Ophiocordycipitaceae were compared, for which consideration was given to the fact that variation in fungal mitogenomes occurs in gene order, genome size, introns, ORFs, and intergenic regions. Mitogenome sizes of nine fungi were significantly different, with the sizes ranging from 23,495 bp to 157,539 bp (Fig. 3; Table 1). Mitogenomic lengths of *Hirsutella rhossiliensis*, *H. vermicola*, *H. minnesotensis*, *H. thompsonii*, *Tolypocladium inflatum*, *T. ophioglossoides*, and *Purpureocillium lilacinum* were an order of magnitude smaller than *O. gracilis* and *O. sinensis*. The mitogenome of *O. sinensis* was the maximum length reported for Ophiocordycipitaceae, which was similar to that of *O. gracilis* (Fig. 3; Table 1). In particular, the introns and non-coding regions were similar in *O. gracilis* and *O. sinensis* (Fig. 1).

The number and size of introns in nine Ophiocordycipitaceae fungi were measured and compared. (1) The largest was *O. sinensis*, which contained 54 introns with a total size of 106,554 bp. (2) *O. gracilis* was the second largest, and contained 49 introns totaling 82,380 bp. (3) *H. minnesotensis* contained 13 introns totaling 17,123 bp. (4) *H. thompsonii* contained 13 introns totaling 23,972 bp. (5) *H. rhossiliensis* had 9 introns totaling 12,477 bp. (6) *H. vermicola* had 7 introns totaling 15,549 bp. (7) *T. ophioglossoides* had 6 introns totaling 7913 bp. (8) *T. inflatum* had 1 intron of 1690 bp. (9) *P. lilacinum* was the smallest and contained, 1 intron totaling 1638 bp (Table 1).

The synteny results in Mauve revealed that the homologous collinear blocks occurred in all nine fungal mitogenomes of Ophiocordycipitaceae (Fig. 3). An observation could be made that nine species were highly similar in PCGs, ribosomal RNA, and tRNA gene composition and quantity. The 15 PCGs and 2 rRNA genes of the nine species were arranged as rnl (rps3) -nad2 -nad3 -atp9 -cox2 -nad4L -nad5 -cob -cox1 -nad1 -nad4 -atp8 -atp6 -rns -cox3 -nad6. All genes were arranged in the same order as the gene arrangement of Ophiocordycipitaceae (Additional file 4: Table S3). At the synteny level, the mitogenome of *O. gracilis* was conserved in the gene arrangement and gene order of 15 PCGs, 2 rRNA genes and the majority of tRNA genes of Ophiocordycipitaceae. Owing to the high evolution rate, mitogenomic introns can differ between species within the same genus or even intra-species (Deng et al. 2016). Of note, the mitogenome of *O. sinensis* contains the largest number of introns and intergenic regions, and *O. gracilis* is the second largest. Therefore, the increased size of mitogenomes may be the result of intergenic region expansion.

*Ophiocordyceps gracilis* was similar to *O. sinensis*, and both had gene rearrangements and homologous regions in the mitogenome (Additional files 1: Fig. S1, 2: Table S1). The results of Circoletto analysis indicate that the similarity of mitogenomes of *O. gracilis* and *O. sinensis* was 87% (Fig. S1) with bases containing 51,041 bp. Multiple introns and intergenic regions were present in the mitogenomes of *O. gracilis* and *O. sinensis*, which differed in location, number, and length.

**Fig. 4:**
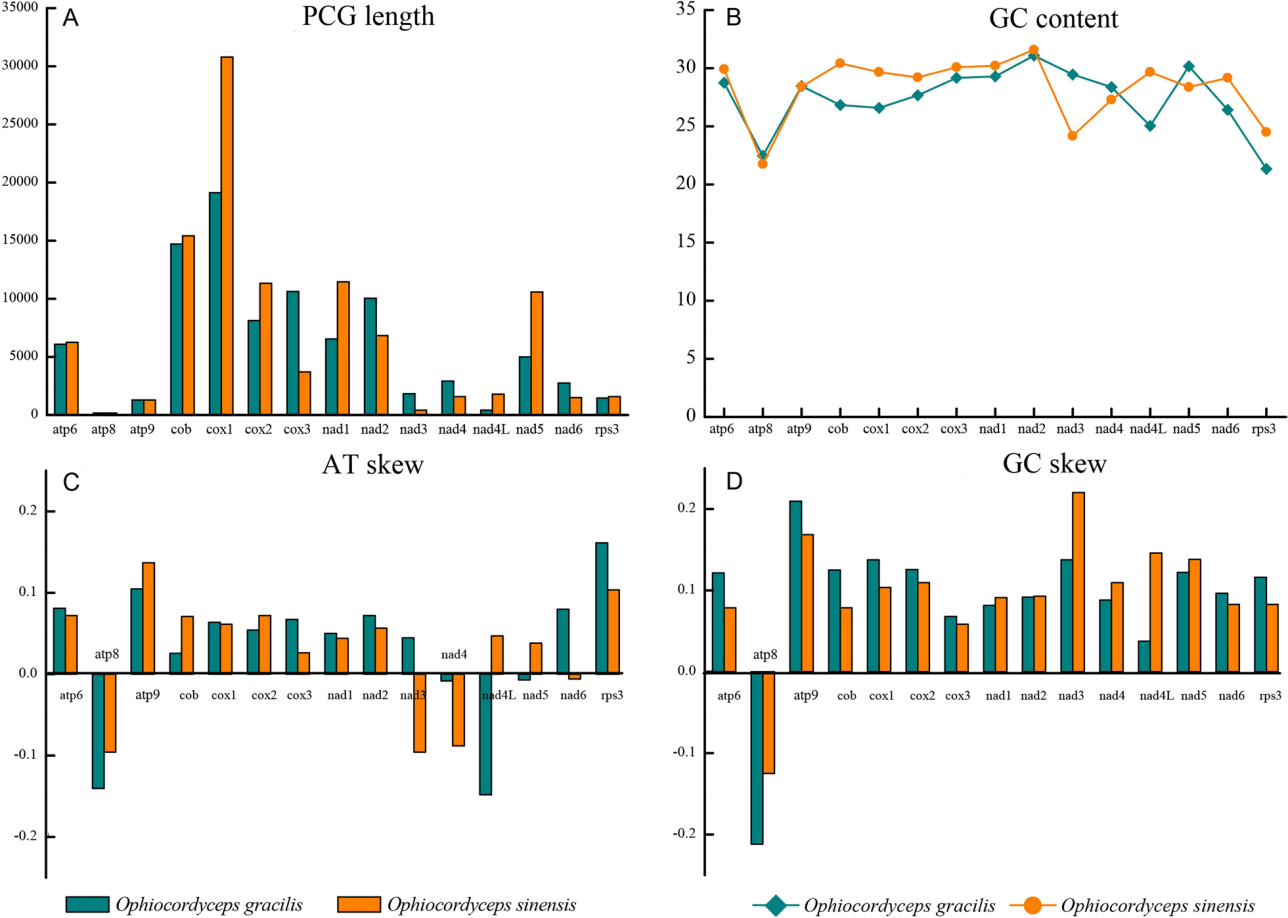
Variation in the length and base composition of each of 15 protein-coding genes (PCGs) between *Ophiocordyceps gracilis* and *O. sinensis* (Kang et al. 2017)*.* **a** PCGs length variation; **b** GC content; **c** AT-stew; **d** GC-stew. Light sea green represents *O. gracilis*, and orange represents *O. sinensis*

An in-depth comparison was conducted on the differences and similarities between *O. gracilis* and *O. sinensis*. Unlike *O. sinensis*, nad2/nad3 genes and nad4L/nad5 in *O. gracilis* were connected, and an overlap of base “A” was also observed (Li et al. 2015; Kang et al. 2017) (Fig. 1; Additional file 2: Table S1). The differences included varying lengths of 15 PCGs across the mitogenomes of *O. gracilis* and *O. sinensis*, in which cox1, cox2, cox3, nad1, nad2, nad3, nad4, nad4L, nad5, and nad6 exhibited differences in length, while atp8 and atp9 were the same (Fig. 4a). The GC content in the *O. gracilis* mitogenome was 28.65%, which was lower than that in the *O. sinensis* mitogenome (30.2%). The GC content of 15 PCGs varied across the mitogenomes. The nad2 contained the highest GC content in both *O. gracilis* and *O. sinensis* (31.09% and 31.58%, respectively) (Fig. 4b). There was a positive AT skew of atp6, atp9, cob, cox1, cox2, cox3, nad1, nad2, nad3, nad6, and rps3, while atp8, nad4, nad4L, and nad5 had a negative A–T skew. Most PCGs exhibited a positive A–T skew in *O. gracilis* and *O. sinensis*. In addition, nad4L and nad5 exhibited a negative A–T skew in *O. gracilis* and a positive A–T skew in *O. sinensis*.

A negative A-T skew was observed in atp8 and nad4 while a positive A-T skew is present in nad3 and nad6 in *O. graciliis *(Fig. 4c). Most PCGs exhibited a positive G–C skew and only atp8 exhibited a negative G-C skew. The GC content of *O. gracilis* was higher than that of *O. sinensis* at atp6, atp8, atp9, cob, cox1, cox2, cox3, nad6, and rps3 (Fig. 4d).

**Fig. 5:**
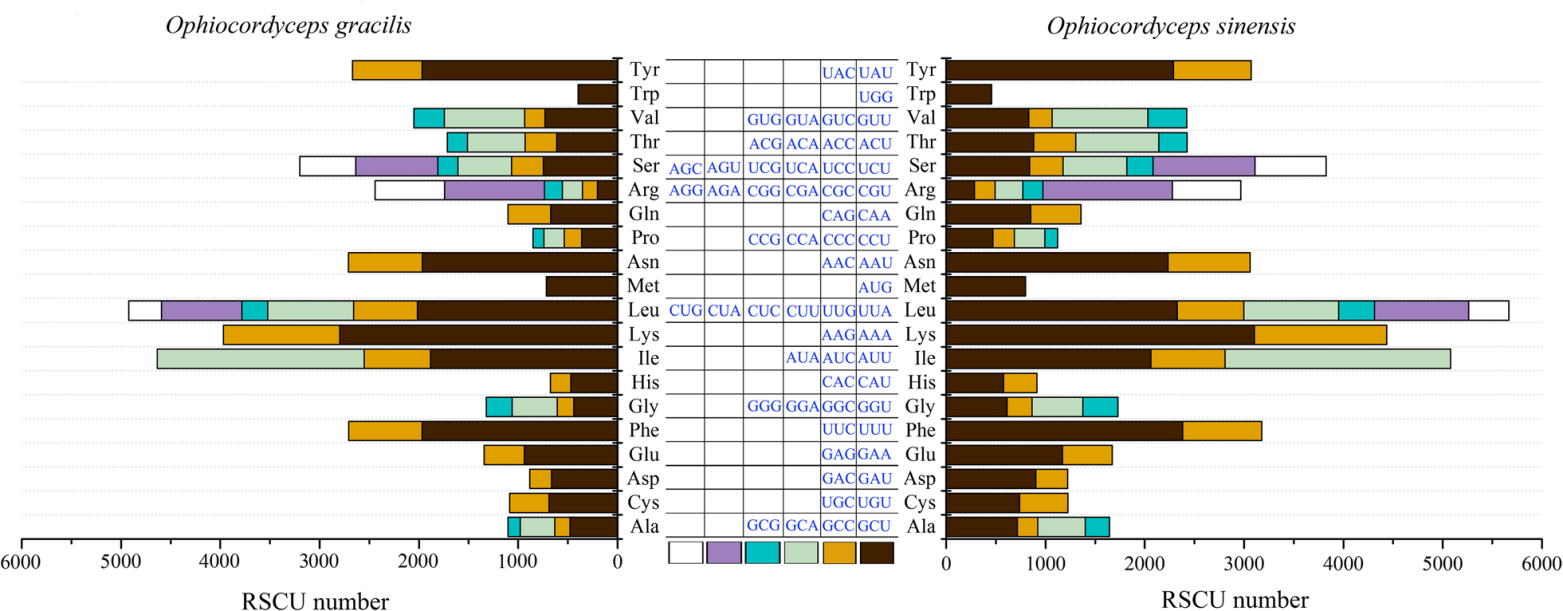
Relative synonymous codon usage (RSCU) in PCGs of *Ophiocordyceps gracilis* and *O. sinensis* (Kang et al. 2017)

The relative synonymous codon usage (RSCU) values of *O. gracilis* and *O. sinensis* are outlined in Fig. 5 and Additional file 5: Table S4. The pattern of codon usage was generally similar among the fungal mitochondria, including the most frequently used codons UUU, UUA, CUU, AUU, AUA, AUG, GUU, GUA, and 30 other codons, except for UAA. Based on the codon usage analysis, the most used codons in the two mitogenomes were UUU (for Phe), AUU and AUA (for IIe), AAA (for Lys), UUA (for Leu), AAU (for Asn), and UAU (for Tyr). The high frequency of A and T use in codons was observed (Additional file 5: Table S4).

Four ORFs were predicted in *O. gracilis*, while 73 ORFs were predicted in *O. sinensis* (Additional file 2: Table S1). The lengths of ORFs were 2256 bp (orf751), 819 bp (orf272), 666 bp (orf221), and 519 bp (orf172). The mitogenome of *O. gracilis* demonstrated variation in the relative positions and sizes of ORFs. Conversely, the mitogenome of *O. sinensis* included hypothetical protein (35), LAGLIDADG endonuclease (27), GIY-YIG endonuclease (8), RNA-dependent DNA polymerase (1), RNA polymerase (1), and GIY-YIG/LAGLIDA-DG (1).

The comparative analysis of tRNA genes in the *O. gracilis* and *O. sinensis* mitogenomes show that *O. gracilis* contains two copies of tRNA-Ser and tRNA-Leu, and three copies of tRNA-Arg, while there were two copies of tRNA-Leu, tRNA-Gly, tRNA-Arg, tRNA-IIe, and tRNA-Ser and three copies of tRNA-Met present in *O. sinensis*. These results suggest that one copy of tRNA-Gly, and tRNA-IIe may have been lost in *O. gracilis* or tRNA-Arg was duplicated in *O. sinensis* (Additional file 2: Table S1)*.* Hence, an observation could be made that sequence differences in tRNA genes were associated with variation, which was central to phylogenic and genetic relationship.

### Identification of repetitive elements in *O. gracilis* and *O. sinensis*

Bibiserv-REPuter was applied to detect repeated sequences, it can locate and identify forward, reverse, complementary and palindromic repeats (Kurtz et al. 2001). To compare the differences between *O. gracilis* and *O. sinensis*, the default parameter settings were used, with the maximum computed repeats and minimal repeat size being limited to 50 and 8, respectively. In the mitogenome of *O. gracilis*, REPuter identified 32 forward types (a total of 3998 bp), 11 palindromic types (a total of 1400 bp), and 7 of reverse repeat types (a total of 780 bp). Meanwhile, 32 forward types (a total of 5158 bp), 14 palindromic types (a total of 2064 bp), and 4 reverse repeat types (a total of 556 bp) were identified in *O. sinensis* (Additional file 6: Table S5). The Tandem Repeats Finder was used to detect 48 and 44 tandem repeats in *O. gracilis* and *O. sinensis*, respectively (Additional file 7: Table S6). The longest tandem sequence was observed in the mitogenomes of *O. gracilis* (57 bp) and *O. sinensis* (123 bp). Most tandem repeat sequences were copied in the two species. Tandem repeat sequences accounted for 1.93% and 1.76% of the mitogenome lengths of *O. gracilis* and *O. sinensis*, respectively.

Interspersed repeats and partial tandem repeats (microsatellites and minisatellites) are moderately repetitive sequences. As simple sequence repeats (SSRs) or short tandem repeats (STR), the basic component of a microsatellite is a short sequence motif (1–6 bp in length) repeated in tandem (Li et al. 2018). Microsatellites are ubiquitous and highly variable across all eukaryotic groups. In general, the microsatellite frequency is positively correlated with the genome size (Garner 2002). A microsatellite identification tool detected SSRs numbers of 46 and 43 in *O. gracilis* and *O. sinensis*, respectively (Additional file 8: Table S7)*.* The majority of these SSRs sequences were rich in A/T, and 31 and 36 of A/T were in the mitogenome of *O. gracilis* and *O. sinensis*, respectively. Thus, the rapid expansion in mitogenomes of *O. gracilis* and *O. sinensis* was promoted by repeats and duplicates.

### Expansion of intron in *O. gracilis* and *O. sinensis*

Common in fungal mitochondria and occurring in mitochondrial PCGs and rRNA genes, introns are ideal tools to investigate genetic variation within fungi and to identify different species (Sosa-Gómez et al. 2009; Xu et al. 2009; Qin et al. 2010; Ferandon et al. 2013; Zhang and Zhang 2019). The accumulation, movement, and degeneration of introns can effectuate intronic polymorphism in closely related species on an intraspecific level. The gain and loss of introns in these conserved genes, including rnl and rns, may occur by horizontal gene transfer (Lang et al. 2007). Premised on the secondary RNA structures and splicing mechanism, these introns are primarily categorized into Groups I or II (Saldanha et al. 1993; Lang et al. 2007); Group I introns are relatively more abundant in fungal mitogenomes (Zhang and Zhang 2019).

A total of 49 introns were detected in the *O. gracilis* mitogenome, with 12 in PCGs and 1 site in rRNA (rnl) (Additional file 9: Table S8). In the *O. gracilis* mitogenome, 37 introns were categorized into Group I, 3 were in Group II, and 9 introns were not classified. Within Group I, the introns were further subdivided into IA (with 7 introns), IB (19), IC (8), and ID (3). IB was the largest subgroup, which was also the same in the other fungi (Li et al. 2015; Zhang et al. 2015b; Kang et al. 2017; Wang et al. 2018).

The *O. gracilis* introns were identified in rnl, nad2, nad3, atp9, cox2, nad5, cob, cox1, nad1, nad4, atp6, cox3, and nad6. Introns were most common in cox1 (10 sites) and cob (8 sites); rnl, nad2, and cox3 all had 5 sites; cox2 had 4 sites; nad5 and nad1 had 3 sites; atp6 had 2 sites; nad3, atp9, nad4, and nad6 were only identified in 1 site; and no introns were identified in nad4L, atp8, and rns. In *O. sinensis*, there was no indication of nad3, nad4, and atp8. However, rns was detected across 8 sites in *O. sinensis*. The introns in rnl accounted for 63.78% and PCGs accounted for 84.32%. The introns of *O. gracilis* had an overall length of 82,380 bp and constituted 61.35% of the entire mitogenome (Table 1). Combined with the analysis of *O. gracilis* and several other fungi, an assumption could be made that, for most fungal species, Group I introns may be included in the rps3 gene within the large subunit ribosomal RNA (rnl) gene (Zhang et al. 2017a,b; Wang et al. 2018).

The number, size, gene content, and insertion site vary among species with a close association with each other and also within the same species (Jung et al. 2012; Torriani et al. 2014; Jalalzadeh et al. 2015; Zhang et al. 2015a; Li et al. 2020b). The genomic information analysis indicates that *O. gracilis* is considerably similar to *O. sinensis* in the family Ophiocordycipitaceae (Table 1), where the number and size of introns in *O. gracilis* are revealed to be the second largest, below those in *O. sinensis* (Kang et al. 2017). Therefore, an assumption could be made that *O. gracilis* and *O. sinensis* are significantly different from other fungi of Ophiocordycipitaceae, with large mitogenomes and many introns. Mitogenome comparison demonstrates that intron length significantly contributes to the size of the mitogenome (Himmelstrand et al. 2014; Zhang et al 2015a; Deng et al. 2016). Hence, the intron presence/absence dynamics of the *O. gracilis* and *O. sinensis* mitogenomes can also further the understanding of the evolutionary dynamics of mitochondrial introns.

### Phylogenetic analysis

**Fig. 6:**
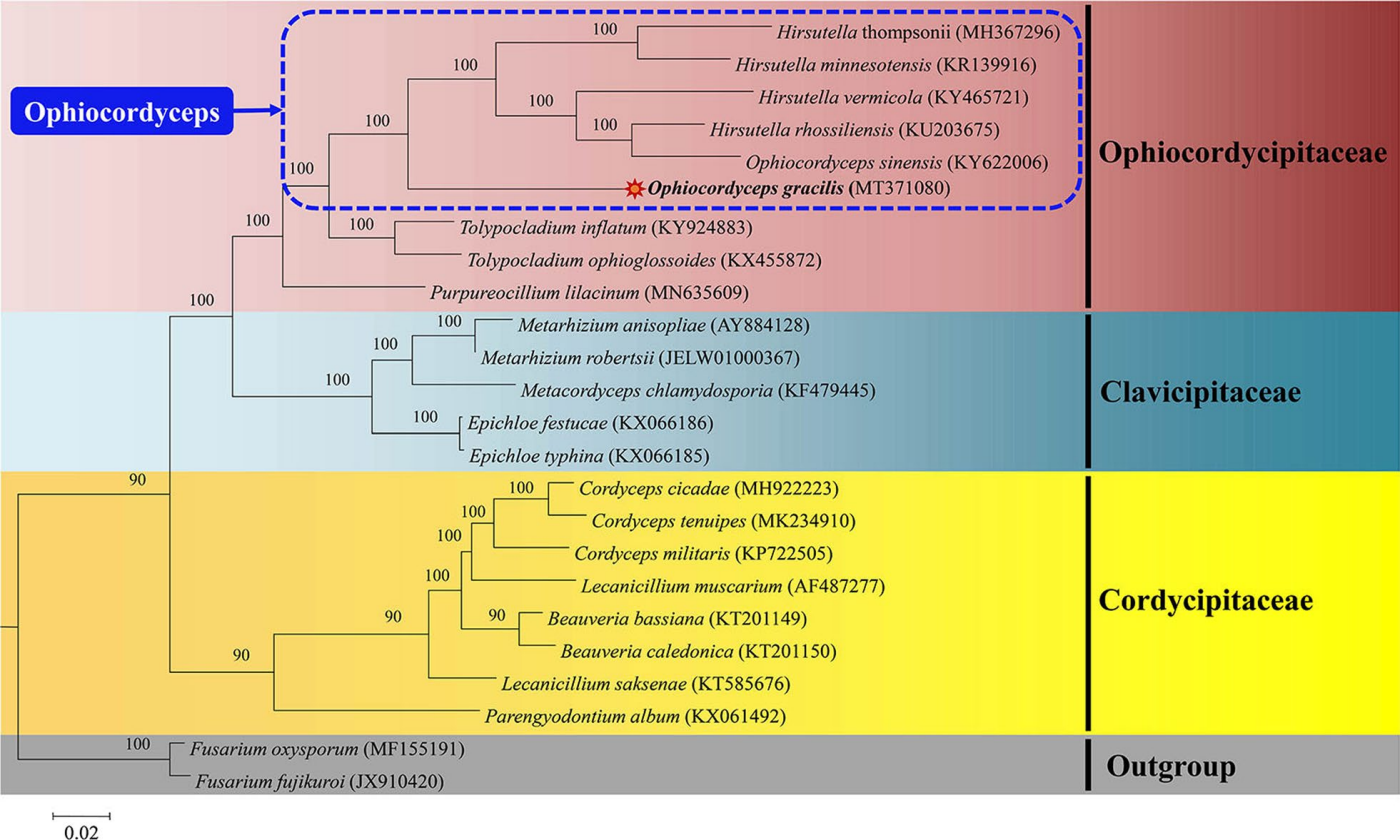
Phylogenetic relationships of *Ophiocordyceps gracilis* and 23 other taxa of Hypocreales inferred from mitogenomes based on the Bayesian inference. *Ophiocordyceps* is framed with a dotted blue line. The species and GenBank accession number for mitogenomes used in the phylogenetic analysis are provided in Additional file 10: Table S9

PCGs and rRNA genes from the mitogenome can also provide useful information to help understand the evolution and phylogeny of fungi (Wang et al. 2018, 2020a). The BI analysis was employed to reveal the phylogenetic position of *O. gracilis* and 23 other species from Hypocreales (Additional file 10: Table S9). On the basis of the combined gene dataset (15 PCGs and 2 rRNA) using the GTR + G + I model (Ronquist et al. 2012), a phylogenetic tree was constructed. All major clades within the tree had sufficient bootstrap support [Bootstrap support (BS) ≥ 90] (Fig. 6). The 24 species of Hypocreales highly varied in size and were divided into three major clades including the families of Ophiocordycipitaceae, Clavicipitaceae, and Cordycipitaceae (Additional file 10: Table S9). The results indicated that *O. gracilis* was clustered in the genus *Ophiocordyceps* of Ophiocordycipitaceae to generate a separate clade with strong statistical support by posterior probabilities (BI-PP = 100%). Because closeness to the *O. sinensis* clade was exhibited, it was suggested that *O. gracilis* and *O. sinensis* originated from a common ancestor. It was surprising to see rps3 included in rnl, both in *O. gracilis* and *O. sinensis*. Of note, for both *O. gracilis* and *O. sinensis*, rps3 is present in rnl, which is different from 14 core PCGs in Hypocreales fungi (Lin et al. 2015; Kang et al. 2017).

To understand the evolution of fungi, mitochondrial genes were adopted in molecular phylogenetics and population genetics research (Nadimi et al. 2016; Dai et al. 2018). Many phylogenetic studies on fungal mitochondria, such as the large subunit gene (rnl), investigate how different organisms are related to each other (Borstler et al. 2008). These analyses seek to identify genes, which provide a clear phylogenetic signal, that differ among closely related taxa and are important in the assessment of the evolutionary relationships of mitogenomes (Nadimi et al. 2016). In this study, the phylogenetic topological structure is consistent with those in prior studies, where inference was made from both BI analysis based on 27 taxa of the Hypocreales order (Li et al. 2019a). Mitochondrial genes are useful molecular markers for determining the phylogenetic relationships in the family Ophiocordycipitaceae.

## CONCLUSION

To our knowledge, this study is the first in which the overall characteristics of the mitogenome of *O. gracilis* was sequenced, assembled, annotated, and analyzed*.* As the second largest reported mitogenome in Ophiocoedycipitaceae, the complete mitogenome of *O. gracilis* was 134,288 bp long, with a non-continuous structure, abundant introns, large intergenic regions, and lack of overlapping genes. The number, length of repeats, duplicates, introns, and PCGs contributed to the size variations in the mitogenome of *O. gracilis*. The genome size, gene content, tRNAs, rRNAs, and collinearity varied among the nine Ophiocordycipitaceae species studied. This difference may indicate that there is variation in introns and intergenic regions. The mitogenome sizes of *O. gracilis* and *O. sinensis* were an order of magnitude higher than those of seven Ophiocordycipitaceae species. In particular, the types and amounts of tRNA, intron, and ORF were significantly different. An observation was made that the mitogenome of *O. sinensis* exhibits similarity to *O. gracilis* in Ophiocordycipitaceae, especially in terms of introns and non-coding regions. The presence/absence of introns suggests that introns are related to the evolutionary dynamics in the mitogenome.

The mitogenomes were compared to verify the phylogenetic relationship between nine related Ophiocordycipitaceous fungi. Using collinear calculation, all nine species were determined to contain 6 homologous collinear blocks, which maintained the conservation of gene orders at the mitogenome level. The results of comparative mitogenomic analysis indicate that the gene order tended to be conserved, with 14 core PCGs genes and rps3 being invariably retained in nine Ophiocordycipitaceae species. The number of genes was different; in particular, several tRNA had two or three copies, while tRNA-Trp and tRNA-Thr were not predicted in *O. gracilis*. Further, *O. gracilis* has tRNA-Sec (UCA), with selenocysteine (Sec) as the 21^st^ amino acid, which may be related to adaptive evolution.

Phylogenetic analysis demonstrates that *O. gracilis* is clustered in the genus *Ophiocordyceps* of Ophiocordycipitaceae. *Ophiocordyceps gracilis* is closely related to *O. sinensis* and exhibits a close evolutionary relationship. This study provides new insights on the genetics, systematics, and evolution of *O. gracilis*. In addition, the comparative analysis of nine fungal mitogenomes will enrich understanding of the mitochondrial evolution of Ophiocordycipitaceae.

## Supplementary Information

**Additional file 1. Figure S1:** Similarity of the *Ophiocordyceps gracilis* and *O. sinensis* mitogenomes. The sequences are connected by differ ent colors according to similar regions and similar lengths, with scored colors show n in the histograms. The blank area between the connecting lines in the figure indicates that the two species do not have any similarity and are currently allowed only an 8.7% identity. Score coloring: blue ≤ 0.25, green ≤ 0.50, orange ≤ 0.75, red > 0.75. Data obtained from Kang et al. (2017).

**Additional file 2. Table S1:** Gene features and organization of the *Ophiocordyceps gracilis* and *O. sinensis* mitogenomes.

**Additional file 3. Table S2:** tRNA feature and organization in the *Ophiocordyceps gracilis* mitogenome.

**Additional file 4. Table S3:** Gene order of the Ophiocordycipitaceae mitogenomes.

**Additional file 5. Table S4:** Codon usage analysis of two different *Ophiocordyceps* species.

**Additional file 6. Table S5:** Distribution of repeat loci in the mitogenome of *Ophiocordyceps gracilis* and *O. sinensis* as revealed by REPuter.

**Additional file 7. Table S6:** Tandem repeats detected in the mitogenome of *Ophiocordyceps gracilis* and *O. sinensis*.

**Additional file 8. Table S7:** Microsatellite DNA in *Ophiocordyceps gracilis* and *O. sinensis* mitogenome.

**Additional file 9. Table S8:** Introns in the *Ophiocordyceps gracilis* mitogenome.

**Additional file 10. Table S9:** Mitogenome information used for phylogenetic analysis.

## Data Availability

All data generated or analyzed during this study are included in this published article. The authors promise the availability of supporting data.

## Abbreviations

Mitogenome: Mitochondrial genome
mtDNA: Mitochondrial DNA
BI: Bayesian inference
ML: Maximum likelihood
FMGP: Fungal mitochondrial genome project
Nr: Non-redundant protein sequence database
PCGs: Protein-coding genes
tRNA: Transfer RNA gene
rRNA: Ribosomal RNA gene
rnl: Large subunit ribosomal RNA
rns: Small subunit ribosomal RNA
orf: Open reading frame
IRs: Intergenic regions
SSRs: Simple sequence repeats
STR: Short tandem repeat
BS: Bootstrap support
F: Forward
R: Reverse
C: Complement
p: Palindromic
PRICE: Paired-read iterative contig extension
SRA: Short read archive
