# Supplementary figures and images for: The complete mitochondrial genome of Ophiocordyceps gracilis and its comparison with related species
Source: IMA Fungus. 2021 Oct 20;12:31. doi: 10.1186/s43008-021-00081-z (PMC8527695; doi:10.1186/s43008-021-00081-z)

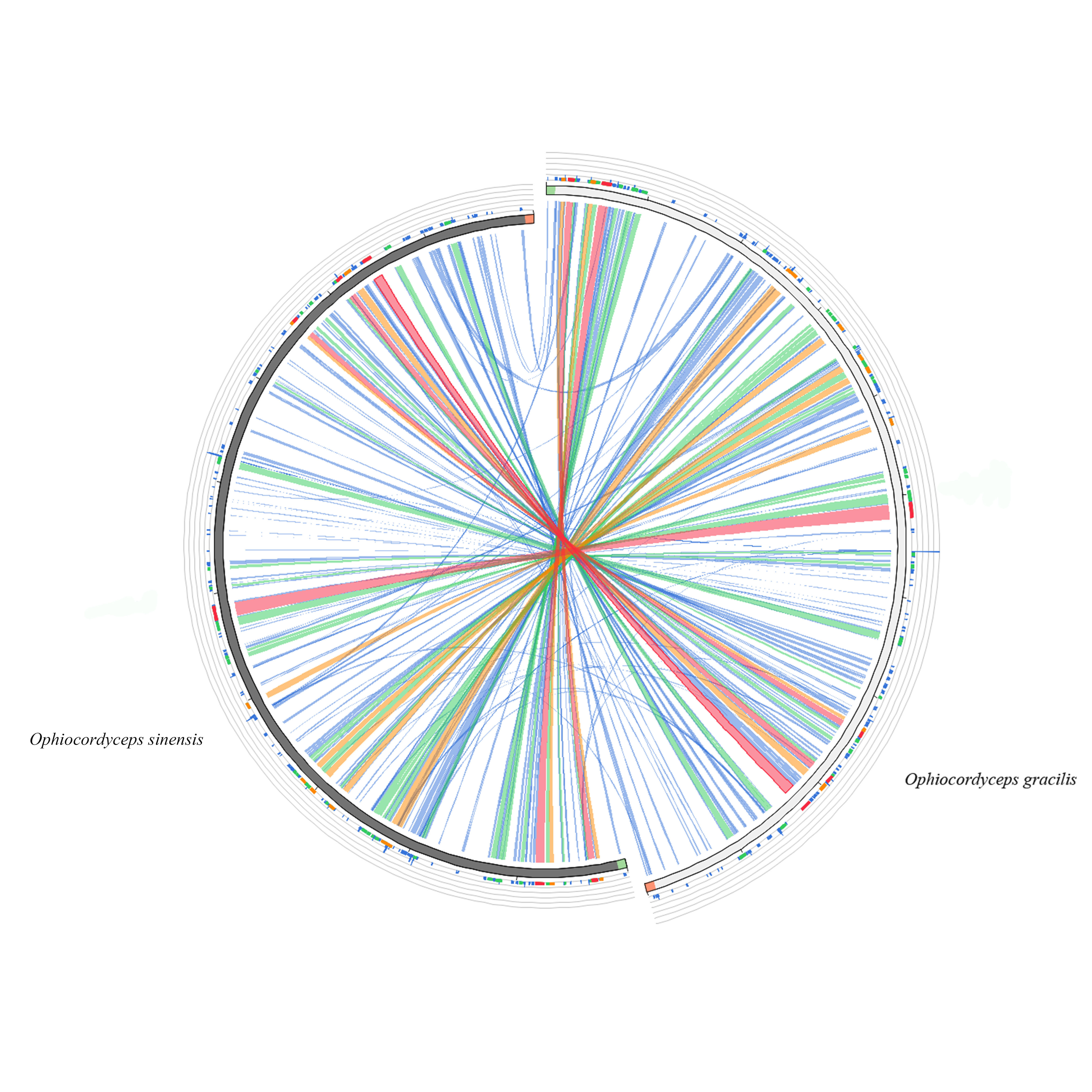

Supplement: Supplementary file 1 — Additional file 1. Figure S1: Similarity of the Ophiocordyceps gracilis and O. sinensis mitogenomes. The sequences are connected by differ ent colors according to similar regions and similar lengths, with scored colors show n in the histograms. The blank area between the connecting lines in the figure indicates that the two species do not have any similarity and are currently allowed only an 8.7% identity. Score coloring: blue ≤ 0.25, green ≤ 0.50, orange ≤ 0.75, red > 0.75. Data obtained from Kang et al. (2017). [file 43008_2021_81_MOESM1_ESM.jpg]
