# Supplementary material for: The complete mitochondrial genome of Ophiocordyceps gracilis and its comparison with related species
Source: IMA Fungus. 2021 Oct 20;12:31. doi: 10.1186/s43008-021-00081-z (PMC8527695; doi:10.1186/s43008-021-00081-z)
Supplement: Supplementary file 3 — Additional file 3. Table S2: tRNA feature and organization in the Ophiocordyceps gracilis mitogenome. [file 43008_2021_81_MOESM3_ESM.doc]

| No. | Feature | Location | Anticodon | Length | strand |
| --- | --- | --- | --- | --- | --- |
| 1 | tRNA-Val | 200-272 | TAC | 73 | + |
| 2 | tRNA-IIe | 1199-1270 | GAT | 72 | + |
| 3 | tRNA-Ser | 1313-1399 | TGA | 87 | + |
| 4 | tRNA-Sec | 1401-1473 | TCA | 73 | + |
| 5 | tRNA-Pro | 1482-1554 | TGG | 73 | + |
| 6 | tRNA-Glu | 16501-16575 | TTC | 75 | + |
| 7 | tRNA--Met | 16659-16731 | CAT | 73 | + |
| 8 | tRNA-Leu | 16732-16814 | TAA | 83 | + |
| 9 | tRNA-Phe | 20989-21061 | GAA | 73 | + |
| 10 | tRNA-Lys | 21160-21232 | TTT | 73 | + |
| 11 | tRNA-Ala | 22288-22360 | TGC | 73 | + |
| 12 | tRNA-Leu | 22596-22681 | TAG | 86 | + |
| 13 | tRNA-Gln | 25339-25411 | TTG | 73 | + |
| 14 | tRNA-His | 25602-25674 | GTG | 73 | + |
| 15 | tRNA-Met | 27990-28063 | CAT | 74 | + |
| 16 | tRNA-Arg | 51157-51229 | ACG | 73 | + |
| 17 | tRNA-Cys | 75161-75235 | GCA | 75 | + |
| 18 | tRNA-Arg | 95150-95224 | TCT | 75 | + |
| 19 | tRNA-Tyr | 117055-117138 | GTA | 84 | + |
| 20 | tRNA-Asp | 117155-117227 | GTC | 73 | + |
| 21 | tRNA-Ser | 117233-117315 | GCT | 83 | + |
| 22 | tRNA-Asn | 118475-118548 | GTT | 74 | + |
| 23 | tRNA-Gly | 130416-130486 | TCC | 71 | + |
| 24 | tRNA-Arg | 130841-130911 | TCG | 71 | + |

**Table S2 tRNA feature and organization in the *O. gracilis* mitogenome**
