# Supplementary material for: The complete mitochondrial genome of Ophiocordyceps gracilis and its comparison with related species
Source: IMA Fungus. 2021 Oct 20;12:31. doi: 10.1186/s43008-021-00081-z (PMC8527695; doi:10.1186/s43008-021-00081-z)
Supplement: Supplementary file 10 — Additional file 10. Table S9: Mitogenome information used for phylogenetic analysis. [file 43008_2021_81_MOESM10_ESM.doc]

**Table S9 Mitogenome information used for phylogenetic analyses**

| **Family** | **Taxon** | **Length** | **GenBank accession** | **Reference** |
| --- | --- | --- | --- | --- |
| Ophiocordycipitaceae | *Hirsutella thompsonii* | 65,332 bp | MH367296 | Wang, *et al.*, 2018 |
| Ophiocordycipitaceae | *Hirsutella minnesotensis* | 52,245 bp | KR139916 | Zhang, *et al.*, 2016 |
| Ophiocordycipitaceae | *Hirsutella vermicola* | 53,793 bp | KY465721 | Zhang, *et al.*, 2017 |
| Ophiocordycipitaceae | *Hirsutella rhossiliensis* | 62,483 bp | KU203675 | Wang, *et al.*, 2016 |
| Ophiocordycipitaceae | *Ophiocordyceps sinensis* | 157,539 bp | KY622006 | Kang, *et al.*, 2017 |
| Ophiocordycipitaceae | *Ophiocordyceps gracilis* | 134,288 bp | MT371080 | This study |
| Ophiocordycipitaceae | *Tolypocladium inflatum* | 24,973 bp | KY924883 | Zhang, *et al.*, 2017 |
| Ophiocordycipitaceae | *Tolypocladium ophioglossoides* | 35,159 bp | KX455872 | Fangliang, *et al.*, 2017 |
| Ophiocordycipitaceae | *Purpureocillium lilacinum* | 23,495 bp | MN635609 | Li, *et al.*, 2020 |
| Clavicipitaceae | *Metarhizium anisopliae* | 24,673 bp | AY884128 | Ghikas, *et al.*, 2016 |
| Clavicipitaceae | *Metarhizium robertsii* | 24,944 bp | JELW01000367 | Unpublished |
| Clavicipitaceae | *Metacordyceps chlamydosporia* | 25,615 bp | KF479445 | Lin, *et al.*, 2015 |
| Clavicipitaceae | *Epichloe festucae* | 88,744 bp | KX066186 | Direct Submission |
| Clavicipitaceae | *Epichloe typhina* | 84,630 bp | KX066185 | Campbell, *et al.*, 2017 |
| Cordycipitaceae | *Cordyceps cicadae* | 56,581 bp | MH922223 | Fan, *et al.*, 2019 |
| Cordycipitaceae | *Cordyceps tenuipes* | 31,386 bp | MK234910 | Li, *et al.*, 2019 |
| Cordycipitaceae | *Cordyceps militaris* | 31,854 bp | KP722505 | Zhang, *et al.*, 2015 |
| Cordycipitaceae | *Lecanicillium muscarium* | 24,499 bp | AF487277 | Kouvelis, *et al.*, 2004 |
| Cordycipitaceae | *Beauveria bassiana* | 29,944 bp | KT201149 | Unpublished |
| Cordycipitaceae | *Beauveria caledonica* | 38,316 bp | KT201150 | Unpublished |
| Cordycipitaceae | *Lecanicillium saksenae* | 25,919 bp | KT585676 | Xin, *et al.*, 2016 |
| Cordycipitaceae | *Parengyodontium album* | 28,081 bp | KX061492 | Yuan, *et al.*, 2017 |
| Nectriaceae | *Fusarium oxysporum* | 46,026 bp | MF155191 | Unpublished |
| Nectriaceae | *Fusarium fujikuroi* | 46,761 bp | JX910420 | Fourie, *et al.*, 2013 |

**References**

Campbell M A, Tapper B A, Simpson W R, Johnson R D, Mace W, Ram A, Lukito Y, Dupon P, Johnson L J, Scott D B, Ganley A R D, Cox M P (2017) *Epichloë hybrida*, sp. nov., an emerging model system for investigating fungal allopolyploidy. Mycologia 109: 715-729. https://doi.org/10.1080/00275514.2017.1406174

Huang F, Li Y, Chen X (2017) The complete mitochondrial genome of a medicinal fungus, *Tolypocladium ophioglossoides*. Mitochondrial DNA Part B 2: 95-96. https://doi.org/10.1080/23802359.2017.1285208

Fan W, Zhang S, Zhang Y (2019) The complete mitochondrial genome of the Chan-hua fungus *Isaria cicadae*: a tale of intron evolution in Cordycipitaceae. Environmental Microbiology 21: 864-879.

https://doi.org/10.1111/1462-2920.14522

Fourie G, Merwe N A V D, Wingfield B D, Bogale M, Tudzynski B, Wing M J, Steenkamp E T (2013) Evidence for inter-specific recombination among the mitochondrial genomes of Fusarium species in the *Gibberella fujikuroi* complex. BMC Genomics. 14: 605. https://doi.org/10.1186/1471-2164-14-605

Ghikas D V, Kouvelis V N, Typas M A (2006) The complete mitochondrial genome of the entomopathogenic fungus *Metarhizium anisopliae* var. *anisopliae:* gene order and *trn* gene clusters reveal a common evolutionary course for all Sordariomycetes, while intergenic regions show variation. Archives of Microbiology 185: 393-401. https://doi.org/10.1007/s00203-006-0104-x

Kang X, Hu L, Shen P, Li R, Liu D (2017) SMRT sequencing Revealed Mitogenome Characteristic and Mitogenome-Wide DNA Modification Pattern in *Ophiocordyceps sinensis*. Frontiers in Microbiology 8: 1422.

https://doi.org/10.3389/fmicb.2017.01422

Kouvelis, V.N., Ghikas, D.V., Typas, M.A., 2004. The analysis of the complete mitochondrial genome of *Lecanicillium muscarium* (synonym Verticillium lecanii) suggests a minimum common gene organization in mtDNAs of Sordariomycetes: phylogenetic implications. Fungal Genetics and Biology 41: 930-940. https://doi.org/10.1016/j.fgb.2004.07.003

Lin R, Liu C, Shen B, Bai M, Ling J, Chen G (2015) Analysis of the complete mitochondrial genome of *Pochonia chlamydosporia* suggests a close relationship to the invertebrate-pathogenic fungi in Hypocreales. BMC Microbiology 15: 5. [https://doi.org/10.1186/s12866-015-0341-8](https://doi.org/10.1186/s12866-015-0341-8.)

Lin R, Liu C, Shen B, Bai M, Ling J, Chen G (2015) Analysis of the complete mitochondrial genome of *Pochonia chlamydosporia* suggests a close relationship to the invertebrate-pathogenic fungi in Hypocreales. BMC Microbiology 15: 5. https://doi.org/10.1186/s12866-015-0341-8

Li J, Zhang G, Yu H, Huang L, Zeng W, Wang Y (2020) Complete mitochondrial genome of the important bio-control fungus *Purpureocillium lilacinum* (Ophiocordycipitaceae, Hypocreales) and its phylogenetic analysis. Mitochondrial DNA Part B 5: 240-242.

https://doi.org/10.1080/23802359.2019.1699466

Wang N, Zhang Y, Hussain M, Li K, Xiang M, Liu X (2016) The mitochondrial genome of the nematode endoparasitic fungus *Hirsutella rhossiliensis*. Mitochondrial DNA Part B 1: 114-115.

https://doi.org/10.1080/23802359.2016.1143336

Wang L, Zhang S, Li J, Zhang Y (2018) Mitochondrial genome, comparative analysis and evolutionary insights into the entomopathogenic fungus *Hirsutella thompsonii*. Environmental Microbiology 20: 3393-3405.

https://doi.org/10.1111/plb.12414

Xin B, Lin R, Shen B, Mao Z, Cheng X, Xie B (2016) The complete mitochondrial genome of the nematophagous fungus *Lecanicillium saksenae*. Mitochondrial DNA Part A 27: 3246-3247. https://doi.org/10.3109/19401736.2015.1110794

Yuan X, Mao X, Liu X, Cheng S, Zhang P, Zhang Z (2017) The complete mitochondrial genome of *Engyodontium album* and comparative analyses with Ascomycota mitogenomes. Genetics and Molecular Biology 40: 844-854. https://doi.org/10.1590/1678-4685-GMB-2016-0308

Zhang Y, Zhang S, Zhang G, Liu X, Wang C, Xu J (2015) Comparison of mitochondrial genomes provides insights into intron dynamics and evolution in the caterpillar fungus *Cordyceps militaris*. Fungal Genetics and Biology 77: 95-107. https://doi.org/10.1016/j.fgb.2015.04.009

Zhang Y, Zhang S, Liu X (2016) The complete mitochondrial genome of the nematode endoparasitic fungus *Hirsutella minnesotensis*. Mitochondrial DNA Part A 27: 2693-2694. https://doi.org/10.3109/19401736.2015.1046126

Zhang Y, Zhang H, Liu X, Zhang S (2017) Mitochondrial genome of the nematode endoparasitic fungus *Hirsutella vermicola* reveals a high level of synteny in the family *Ophiocordycipitaceae*. Applied Microbiology Biotechnology 101: 3295-3304. https://doi.org/10.1080/23802359.2016.1143336

Zhang Y, Yang X, Zhang S, Humber R A, Xu J (2017) Genomic analyses reveal low mitochondrial and high nuclear diversity in the cyclosporin-producing fungus *Tolypocladium inflatum*. Applied Microbiology and Biotechnology 101: 8517-8531. https://doi.org/10.1007/s00253-017-8574-0
